# The modified Sugiura procedure as bridge surgery for liver transplantation: a case report

**DOI:** 10.1186/s13256-015-0522-y

**Published:** 2015-03-04

**Authors:** An-Chieh Feng, Chi-Yang Liao, Hsiu-Lung Fan, Teng-Wei Chen, Chung-Bao Hsieh

**Affiliations:** 1 Division of General Surgery, Department of Surgery, Tri-Service General Hospital, National Defense Medical Center, No.325, Sec. 2, Chenggong Rd., Neihu Dist. Taipei, 11490 Taiwan; 2 Department of Surgery, Song Shan Branch, Tri-Service General Hospital, National Defense Medical Center, 131 Jiankang Road, Taipei, 105 Taiwan

**Keywords:** Sugiura procedure, Liver transplantation, Variceal bleeding, Portal hypertension, Liver cirrhosis

## Abstract

**Introduction:**

Esophagogastric varices bleeding is a common complication due to portal hypertension in patients with liver cirrhosis. With the advancement of nonoperative management including vasoactive agents, endoscopic hemostasis or transjugular intrahepatic portosystemic shunt, surgical management has played a lesser role in recent decades. The present report describes a patient with **hepatitis B** (HBV)-related liver cirrhosis and portal vein thrombosis with recurrent esophagogastric varices bleeding despite the use of medical and endoscopic therapy. The modified Sugiura procedure was performed as an alternative bridge surgery for liver transplantation in order not to change the anatomic structure of the great vessels and to avoid hepatic encephalopathy related to shunting procedures like the transjugular intrahepatic portosystemic shunt.

**Case presentation:**

A 56-year-old Chinese man with a history of portal hypertension due to HBV-related liver cirrhosis and known former recurrent esophageal varices bleeding status post Sengstaken-Blakemore tube tamponade was referred to our hospital for liver transplantation evaluation because of persistent esophagogastric varices bleeding with hypovolemic shock, even after medical and endoscopic therapies in a local hospital. As a result, liver cirrhosis with Child-Pugh class B function was diagnosed. Despite the use of vasoactive agents, and endoscopic hemostasis management, esophagogastric varices bleeding still occurred episodically with hypovolemic shock, which could not be reversed by blood transfusion or Sengstaken-Blakemore tube tamponade. The modified Sugiura procedure, as an alternative bridge therapy for patients who are candidates for liver transplantation, was performed, despite the fact that his liver transplantation was not yet completed. He then received a living donor liver transplantation with the right lobe of liver from his daughter. The postoperative course was uneventful, and he was discharged two weeks later. He had no evidence of recurrent esophagogastric varices bleeding during the six-month follow-up.

**Conclusions:**

The treatment experience of this case gave us not only the idea but also the practical way of applying the modified Sugiura operation as a bridge and rescue therapy without alteration of the vascular anatomy and hemodynamic stability for patients who have experienced refractory esophagogastric varices bleeding, despite the use of medication and endoscopic treatment, and are candidates for receiving a liver transplantation.

## Introduction

Gastrointestinal bleeding associated with portal hypertension occurs most commonly from esophageal or gastric varices. Managing variceal bleeding in patients with cirrhosis is most often achieved by medication with vasoactive agents or endoscopic hemostasis or both. However, persistent esophagogastric varices (EV) bleeding occurs in some patients with conditions like coagulopathy, portal vein thrombosis, peptic ulcer disease, and the advanced stage of liver failure. Therefore, some patients with frequently recurrent variceal bleeding need more aggressive therapy such as a transjugular intrahepatic portosystemic shunt (TIPS) or surgical intervention [1].

We describe a patient with hepatitis B (HBV)-related liver cirrhosis and recurrent EV bleeding even after medical and endoscopic management. In order to avoid anatomic and hemodynamic changes and hepatic encephalopathy related to shunting procedures, the modified Sugiura procedure was performed as a rescue therapy and short-term bridge for liver transplantation. The literature was reviewed, and the utility of a modified Sugiura procedure as an alternative bridge therapy for patients who are candidates for liver transplantation is discussed.

## Case presentation

**Figure 1:**
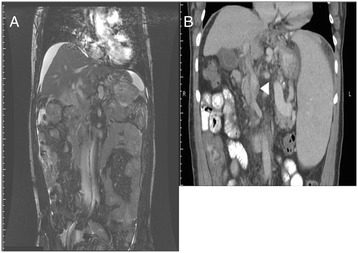
**Preoperative imaging study of the abdomen. (A)** A magnetic resonance imaging scan showed the relatively small size of the portal vein in both hepatic lobes, which may have been due to the hepatofugal flow or stasis flow causing thrombosis. **(B)** A computed tomography scan done in the previous hospital revealed the presence of a long segment of portal vein thrombosis (white arrowhead).

A 56-year-old Chinese man with a history of portal hypertension due to HBV-related liver cirrhosis and known former recurrent esophageal varices bleeding status post Sengstaken-Blakemore tube tamponade was referred to our hospital for liver transplantation evaluation because of persistent EV bleeding with hypovolemic shock, even after medical and endoscopic therapies in a local hospital. On admission, he was hemodynamically unstable and was transferred to the intensive care unit for close monitoring. Grade I encephalopathy and mild ascites were noted during a physical examination, and the serum laboratory tests disclosed hemoglobin 10.2g/dL, total bilirubin 3.3mg/dL, albumin 3.4g/dL, ammonia 220ug/dL, and a prothrombin time of 33.6 seconds. As a result, liver cirrhosis with Child-Pugh class B function was diagnosed. An upper gastrointestinal endoscopy revealed F2 esophageal varices with a healed ulcer, M/3 to L/3, and a magnetic resonance imaging (MRI) scan showed the relatively small size of the portal vein in both hepatic lobes, which may have been due to the hepatofugal flow or stasis flow causing thrombosis (Figure 1A). Besides, a computed tomography (CT) scan done in the previous hospital revealed the presence of a long segment of portal vein thrombosis (Figure 1B).

**Figure 2:**
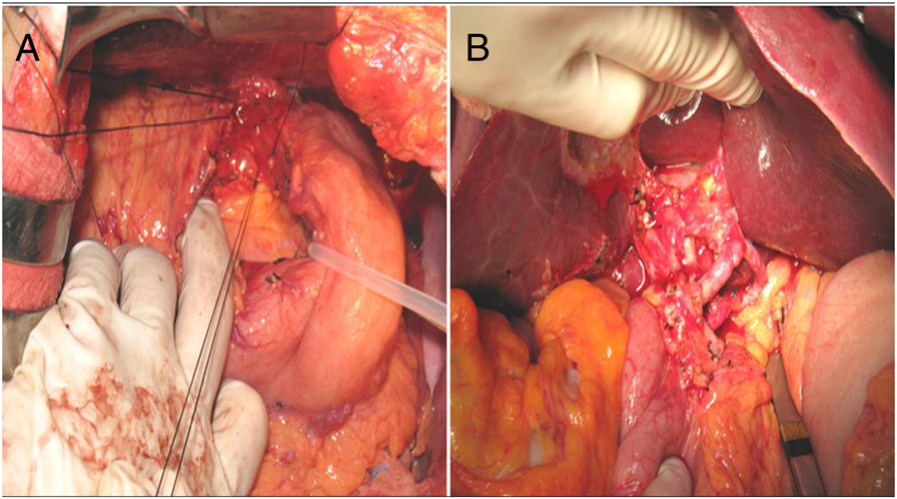
**Intraoperative image. (A)** The modified Sugiura procedure we performed was completed with the devascularization of paraesophagogastric and **(B)** proximal gastric vessels.

Despite the use of vasoactive agents, and endoscopic hemostasis management, EV bleeding still occurred episodically with hypovolemic shock, which could not be reversed by blood transfusion or Sengstaken-Blakemore tube tamponade. Although liver transplantation was the optimal choice for our patient, he and his family still needed some time to consider and the evaluation for his liver transplantation was not yet completed either. As a result, the modified Sugiura operation was performed as an alternative treatment. In surgery, splenomegaly of about 22×12×9cm^3^ in size and engorgement of the vessels over the esophagogastric junction were found. Splenectomy was performed first and the operation was completed with the subsequent devascularization of paraesophagogastric and proximal gastric vessels with a total blood loss of 850mL (Figure 2).

His EV bleeding was under control after the operation, and 11 days later, all the necessary documentation was completed and approved by the medical ethics committee. He then received a living donor liver transplantation with the right lobe of liver (450gm) from his daughter. During the operation, he was found to have portal vein thrombosis, which was compatible with the CT and MRI findings and thrombectomy was performed with a total blood loss of 400cc. The postoperative course was uneventful, and he was discharged two weeks later. He had no evidence of recurrent EV bleeding during the six-month follow-up.

## Discussion

With the advancement of vasoactive agents, endoscopic hemostasis, and TIPS, the need for surgical treatment for EV has decreased. However, in some complicated cases like coagulopathy, portal vein thrombosis, simultaneous peptic ulcer and severe liver cirrhosis, recurrent EV bleeding can be a great challenge for the clinical doctors. Although liver transplantation is considered as the optimal treatment modality for advanced liver disease, refractory EV bleeding may cause rapid deterioration; therefore, emergent surgical interventions, such as shunting or nonshunting procedures, become the options for bleeding control temporally to final transplantation. The frequently applied shunting procedures include splenorenal and mesocaval shunt; for nonshunting procedures, devascularization and splenectomy are the most common.

Sugiura procedure is the nonshunting procedure for EV bleeding, which was first proposed by Sugiura and Futagawa in 1973 [2]. However, because of its complexity and high postoperative morbidity and mortality, this procedure has not been widely accepted in Western countries [3]. Later in the 1980s, the modified Sugiura procedure was introduced and proved to be effective in controlling EV bleeding without complex procedures needed [4]. However, with the development of multimodality therapies, surgical management for controlling EV bleeding is still considered less important, especially in hospitals with the TIPS facility, which have reported promising results previously [5,6]. Nevertheless, obstruction and encephalopathy rates are high [7] and, because of its high clinical technique requirements, TIPS is not available in every hospital. In Taiwan, the TIPS procedure is not covered by the National Health Insurance; therefore, not all patients can afford it. In the present case, we discussed the option of using a TIPS with the patient’s family; however, they refused the procedure because of poor financial status. Alternatively, nonshunting procedures comprising devascularization and splenectomy become another choice for patients with refractory EV bleeding due to cirrhosis of the liver or diffused portal vein thrombosis with less change to the vascular anatomy. We decided to perform a nonshunting procedure based on the following reasons: (1) the unstable preoperative status of our patient, and the fact that shunting procedures may have prolonged the operative time; (2) the shunting procedures would have caused great hemodynamic change and are also related to higher incidence of postoperative hepatic encephalopathy, which may have worsened his pretransplant condition; and (3) shunting procedures may have increased the technical difficulty of the subsequent transplantation and also the related risk of surgical complications.

In recent years, the modified Sugiura operation was also considered to play some role in serving as a short-term bridge therapy for patients with late-stage liver disease who are candidates for liver transplantation. For those patients who have advanced liver failure, liver transplantation is the optimal choice, and shunting or nonshunting operations can be considered as the treatment for bleeding complications. Compared with the risk of postoperative hepatic encephalopathy in surgical shunts and TIPS, nonshunting procedures maintain portal perfusion and hepatic, systemic hemodynamics in patients with cirrhosis, which result in a low incidence of postoperative encephalopathy and many patients have a better quality of life with prolonged survival [8,9].

The modified Sugiura procedure can be performed through a one-stage transabdominal approach via the midline incision or extension of a left subcostal incision with the exposure of an L shape. The procedure starts with splenectomy for improvement of the exposure followed by gastric and esophageal devascularization and finally the esophageal transaction using a mechanical stapler through a short gastrotomy. The Sugiura operation contains five componential procedures and esophagogastric devascularization is the only remaining part in the many different versions of the modified Sugiura operation. The procedures we performed on this patient included esophagogastric devascularization, and splenectomy, and highly selective vagotomy with a total operation time of four hours.

In the early era, operations for managing EV bleeding were considered as a contraindication for patients who were candidates for liver transplantation because they could cause significant upper abdominal adhesions, which might relate to increased intraoperative bleeding and changes of anatomical structures and would result in difficulties with the subsequent transplantation. However, in recent decades, because of the advancement of adhesion-proof materials and the nature of nonshunting operations that do not alter vascular anatomy and hemodynamic stability, these nonshunting operations are considered to become a short-term bridge therapy to liver transplantation without the complication of postoperative severe adhesion, especially for patients with splanchnic thrombosis-caused refractory EV bleeding or to earn more time for the patient and family to consider liver transplantation and also to complete the preoperative evaluations [3,8-11]. Besides, if the patient refused to have a liver transplantation eventually, the modified Sugiura operation could also serve as a long-term bleeding control management with less incidence of encephalopathy and fewer hemodynamic changes [8,9,12].

## Conclusions

The treatment experiences of this case give us not only the idea but also a practical way of applying the modified Sugiura operation as a bridge and rescue therapy without alteration of the vascular anatomy and hemodynamic stability for patients who experience refractory EV bleeding despite the use of medication and endoscopic treatment and are candidates for receiving liver transplantation.

## Consent

Written informed consent was obtained from the patient for publication of this case report and any accompanying images. A copy of the written consent is available for review by the Editor-in-Chief of this journal.

## Abbreviations

EV: Esophagogastric varices
TIPS: Transjugular intrahepatic portosystemic shunt
MRI: Magnetic resonance imaging
CT: Computed tomography
